# 4-(3-Methoxy­phen­oxy)butyric acid

**DOI:** 10.1107/S1600536809008186

**Published:** 2009-03-19

**Authors:** Julia Heilmann-Brohl, Gérard Jaouen, Michael Bolte

**Affiliations:** a École Nationale Supérieure de Chimie de Paris, Laboratoire Charles Friedel, UMR CNRS 7223, 11 rue Pierre et Marie Curie, 75231 Paris Cedex 05, France; b Institut für Anorganische Chemie, J. W. Goethe-Universität Frankfurt, Max-von-Laue-Strasse 7, 60438 Frankfurt/Main, Germany

## Abstract

In the title compound, C_11_H_14_O_4_, an inter­mediate for the synthesis of a new kind of estrogen receptor modulator, all non-H atoms lie on a common plane (r.m.s. deviation = 0.0472 Å). All C—C bonds in the side chain are in a *trans* conformation, and the hydroxyl group is also *trans* to the methyl­ene chain. In the crystal structure, mol­ecules form centrosymmetric dimers showing a head-to-head arrangement which is stabilized by O—H⋯O hydrogen bonds. A weak C—H⋯O contact is also present.

## Related literature

For the synthesis of 4-(3-meth­oxy-phen­oxy)-butyric acid, see Tandon *et al.* (1990 ▶). For estrogen receptor modulators, see Lloyd *et al.* (2004 ▶). For a similar carboxylic acid, see: Smith *et al.* (1989 ▶).
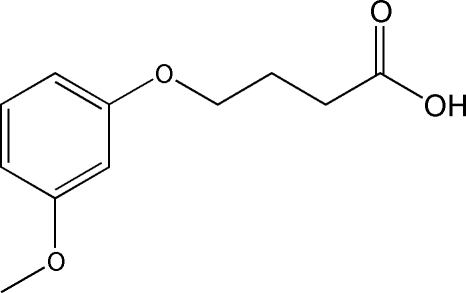


## Experimental

### 

#### Crystal data

C_11_H_14_O_4_
*M* *_r_* = 210.22
Monoclinic, 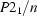

*a* = 9.6509 (6) Å
*b* = 5.3998 (4) Å
*c* = 20.2033 (13) Å
β = 90.822 (5)°
*V* = 1052.74 (12) Å^3^
*Z* = 4
Mo *K*α radiation
μ = 0.10 mm^−1^
*T* = 173 K
0.32 × 0.27 × 0.25 mm

#### Data collection

Stoe IPDS-II two-circle diffractometer
Absorption correction: none
15489 measured reflections
2945 independent reflections
2458 reflections with *I* > 2σ(*I*)
*R*_int_ = 0.057

#### Refinement

*R*[*F* ^2^ > 2σ(*F* ^2^)] = 0.043
*wR*(*F* ^2^) = 0.120
*S* = 1.07
2945 reflections
142 parameters
H atoms treated by a mixture of independent and constrained refinement
Δρ_max_ = 0.31 e Å^−3^
Δρ_min_ = −0.20 e Å^−3^

### 

Data collection: *X-AREA* (Stoe & Cie, 2001 ▶); cell refinement: *X-AREA*; data reduction: *X-AREA*; program(s) used to solve structure: *SHELXS97* (Sheldrick, 2008 ▶); program(s) used to refine structure: *SHELXL97* (Sheldrick, 2008 ▶); molecular graphics: *XP* in *SHELXTL-Plus* (Sheldrick, 2008 ▶) and *Mercury* (Macrae *et al.*, 2006 ▶); software used to prepare material for publication: *SHELXL97* and *PLATON* (Spek, 2009 ▶).

## Supplementary Material

Crystal structure: contains datablocks I, global. DOI: 10.1107/S1600536809008186/ng2557sup1.cif

Structure factors: contains datablocks I. DOI: 10.1107/S1600536809008186/ng2557Isup2.hkl

Additional supplementary materials:  crystallographic information; 3D view; checkCIF report

## Figures and Tables

**Table 1:** Hydrogen-bond geometry (Å, °)

*D*—H⋯*A*	*D*—H	H⋯*A*	*D*⋯*A*	*D*—H⋯*A*
O41—H41⋯O42^i^	0.927 (18)	1.804 (19)	2.7292 (11)	175.5 (16)
C17—H17*B*⋯O42^ii^	0.98	2.48	3.2477 (14)	135

Symmetry codes: (i) 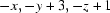; (ii) 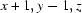.

**Fig. 1.**
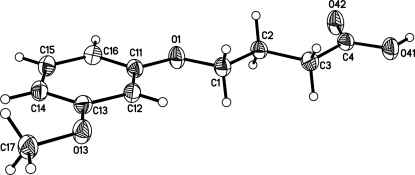
Perspective view of the title compound with the atom numbering scheme; displacement ellipsoids are at the 50% probability level; H atoms are drawn as small spheres of arbitrary radii.

**Fig. 2.**
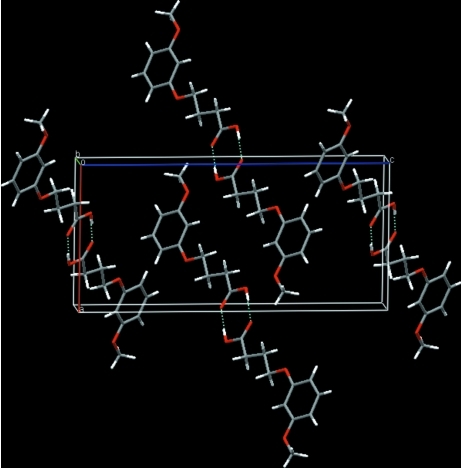
Packing diagram of the title compound with view onto the ac plane. Hydrogen bonds shown as dashed lines.

**Fig. 3.**
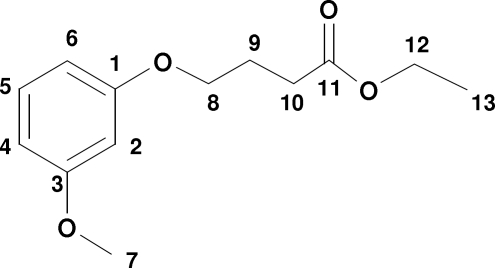
The numbering of the ethyl ester of the title compound.

**Fig. 4.**
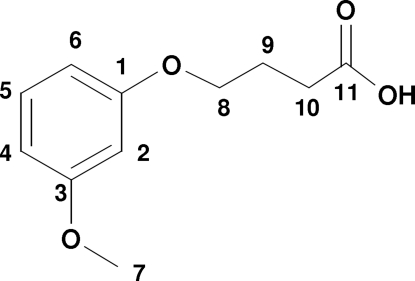
The numbering of the title compound.

## supplementary crystallographic information

### Comment

4-(3-Methoxyphenoxy)butyric acid is an intermediate for the synthesis of a new kind of estrogen receptor modulators (Lloyd *et al.*, 2004). All non-H atoms of the title compound (Fig. 1) lie in a common plane (r.m.s. deviation 0.0472 Å). All C—C bonds in the side chain are in a *trans* conformation, and the hydroxyl group is also *trans* to the methylene chain. In the crystal, the molecules form centrosymmetric dimers showing a head-to-head arrangement which is stabilized by O—H···O hydrogen bonds (Fig. 2). In addition to this classical hydrogen bond, there is weak C—H···O contact (Table 1).

Two comparable structures, 4-(4-chlorophenoxy)butanoic acid and 4-(2,4-dichlorophenoxy)butanoic acid, (Smith *et al.*, 1989) adopt a very similar conformation as the title compound. However, the carboxyl group in these structures is slightly twisted out of the molecular plane. The HO—C(O)—CH_2_—CH_2_ torsion angle is 161.6° and 170.1° in 4-(4-chlorophenoxy)butanoic acid and 4-(2,4-dichlorophenoxy)butanoic acid, respectively, whereas this torsion angle amounts to 174.73 (9)° in the title compound.

### Experimental

Synthesis of 4-(3-methoxy-phenoxy)-butyric acid ethyl ester (scheme 2):

Cs_3_CO_3_ (9.666 mmol, 3.149 g) was added to a solution of 3-methoxyphenol (8.055 mmol, 1.000 g) in acetone (20 ml) and the mixture was stirred for 5 min at r.t.. Ethyl-4-bromobutyrate (8.055 mmol, 1.571 g) was added and the reaction mixture was heated under reflux for 28 h. After cooling to r.t. the slurry was poured onto H_2_O/ice/HCl and the aqeous phase was extracted with CH_2_Cl_2_ (4 *x* 25 ml). The combined organic layers were washed with H_2_O (3 *x* 25 ml), dried over MgSO_4_ and the solvent was removed under reduced pressure to yield the crude product as a slightly yellow oil. The crude product was subjected to a column chromatography (eluent 100% CH_2_Cl_2_), to obtain the pure product as a slightly yellow oil (1.486 g, 77%). ^1^H-NMR (CDCl_3_, 300 MHz): δ = 7.165 (tr, *J* = 8.1 Hz, 1H, C_6_*H*_4_), 6.519 – 6.447 (m, 3H, C_6_*H*_4_), 4.146 (q, *J* = 7.2 Hz, 2H, H12), 3.987 (tr, *J* = 6.2 Hz, 2H, H^8^), 3.780 (s, 3H, O—CH_3_), 2.509 (tr, *J* = 7.2 Hz, 2H H^10^), 2.145 - 2.055 (m, 2H H^9^), 1.259 (tr, *J* = 7.2 Hz, 3H, H^13^).

Synthesis of 4-(3-methoxy-phenoxy)-butyric acid (scheme 3):

4-(3-methoxy-phenoxy)-butyric acid ethyl ester (2.938 mmol, 0.700 g) is dissolved in acetone (10 ml) and H_2_O (5 ml) and 1 *M* NaOH (20 ml) is added. The reaction mixture is stirred at r.t. for 1 h and is then poured into H_2_O/HCl (50 ml). The aqeous phase is extracted with CH_2_Cl_2_ (4 *x* 25 ml), and the combined organic layers are washed with H_2_O (2 *x* 30 ml), dried over MgSO_4_ and the solvent is evaporated. The crude product is obtained as light yellow oil from which colourless crystals – suitable for X-Ray analysis - start to grow within 30 min. Purification of the crude product is conducted by column chromatography. The by-products are removed by elution with CH_2_Cl_2_. The desired product is then eluted with MeOH. After evaporation of MeOH, the pure product is obtained as an off-white crystalline solid (0.352 g, 58%). ^1^H-NMR (CDCl_3_, 300 MHz): δ = 7.171 (tr, *J* = 8.3 Hz, 1H, C_6_*H*_4_), 6.526 - 6.447 (m, 3H, C_6_*H*_4_), 4.010 (tr, *J* = 6.0 Hz, 2H, H^8^), 3.787 (s, 3H, O—CH_3_), 2.592 (tr, *J* = 7.4 Hz, 2H, H^10^), 2.174 - 2.073 (m, 2H, H^9^), n.o. (COO*H*).

### Refinement

H atoms bonded to C were refined with fixed individual displacement parameters [U(H) = 1.2 *U*_eq_(C) or U(H) = 1.5 *U*_eq_(C_methyl_)] using a riding model with C_aromatic_—H = 0.95 Å, C_methyl_—H = 0.98 Å, and C_methylene_—H = 0.99 Å. The methyl group was allowed to rotate but not to tip. the hydroxy H atom was freely refined.

### Crystal data

**Table:**

C_11_H_14_O_4_	*F*(000) = 448
*M**_r_* = 210.22	*D*_x_ = 1.326 Mg m^−^^3^
Monoclinic, *P*2_1_/*n*	Mo *K*α radiation, λ = 0.71073 Å
Hall symbol: -P 2yn	Cell parameters from 15224 reflections
*a* = 9.6509 (6) Å	θ = 3.7–29.5°
*b* = 5.3998 (4) Å	µ = 0.10 mm^−^^1^
*c* = 20.2033 (13) Å	*T* = 173 K
β = 90.822 (5)°	Block, colourless
*V* = 1052.74 (12) Å^3^	0.32 × 0.27 × 0.25 mm
*Z* = 4	

### Data collection

**Table:**

Stoe IPDS-II two-circle diffractometer	2458 reflections with *I* > 2σ(*I*)
Radiation source: fine-focus sealed tube	*R*_int_ = 0.057
graphite	θ_max_ = 29.6°, θ_min_ = 3.7°
ω scans	*h* = −13→13
15489 measured reflections	*k* = −7→7
2945 independent reflections	*l* = −28→25

### Refinement

**Table:**

Refinement on *F*^2^	Secondary atom site location: difference Fourier map
Least-squares matrix: full	Hydrogen site location: inferred from neighbouring sites
*R*[*F*^2^ > 2σ(*F*^2^)] = 0.043	H atoms treated by a mixture of independent and constrained refinement
*wR*(*F*^2^) = 0.120	*w* = 1/[σ^2^(*F*_o_^2^) + (0.0677*P*)^2^ + 0.1161*P*] where *P* = (*F*_o_^2^ + 2*F*_c_^2^)/3
*S* = 1.07	(Δ/σ)_max_ = 0.001
2945 reflections	Δρ_max_ = 0.31 e Å^−^^3^
142 parameters	Δρ_min_ = −0.20 e Å^−^^3^
0 restraints	Extinction correction: *SHELXL97* (Sheldrick, 2008), Fc^*^=kFc[1+0.001xFc^2^λ^3^/sin(2θ)]^-1/4^
Primary atom site location: structure-invariant direct methods	Extinction coefficient: 0.048 (5)

### Special details

**Table:**

Geometry. All e.s.d.'s (except the e.s.d. in the dihedral angle between two l.s. planes) are estimated using the full covariance matrix. The cell e.s.d.'s are taken into account individually in the estimation of e.s.d.'s in distances, angles and torsion angles; correlations between e.s.d.'s in cell parameters are only used when they are defined by crystal symmetry. An approximate (isotropic) treatment of cell e.s.d.'s is used for estimating e.s.d.'s involving l.s. planes.
Refinement. Refinement of *F*^2^ against ALL reflections. The weighted *R*-factor *wR* and goodness of fit *S* are based on *F*^2^, conventional *R*-factors *R* are based on *F*, with *F* set to zero for negative *F*^2^. The threshold expression of *F*^2^ > σ(*F*^2^) is used only for calculating *R*-factors(gt) *etc*. and is not relevant to the choice of reflections for refinement. *R*-factors based on *F*^2^ are statistically about twice as large as those based on *F*, and *R*- factors based on ALL data will be even larger.

### Fractional atomic coordinates and isotropic or equivalent isotropic displacement parameters (Å^2^)

**Table:**

	*x*	*y*	*z*	*U*_iso_*/*U*_eq_	
O1	0.32321 (7)	0.55314 (15)	0.64594 (4)	0.03035 (19)	
C1	0.36009 (11)	0.72446 (18)	0.59488 (5)	0.0263 (2)	
H1A	0.3840	0.6344	0.5539	0.032*	
H1B	0.4411	0.8248	0.6091	0.032*	
C2	0.23475 (10)	0.89015 (19)	0.58265 (5)	0.0265 (2)	
H2A	0.2151	0.9860	0.6232	0.032*	
H2B	0.1527	0.7866	0.5721	0.032*	
C3	0.26116 (11)	1.0679 (2)	0.52546 (5)	0.0286 (2)	
H3A	0.3491	1.1572	0.5343	0.034*	
H3B	0.2727	0.9704	0.4844	0.034*	
C4	0.14701 (11)	1.25525 (19)	0.51430 (5)	0.0269 (2)	
O41	0.17803 (9)	1.41971 (15)	0.46803 (4)	0.0342 (2)	
H41	0.1036 (18)	1.526 (3)	0.4617 (8)	0.056 (5)*	
O42	0.03810 (8)	1.25686 (16)	0.54438 (5)	0.0382 (2)	
C11	0.42179 (10)	0.38789 (18)	0.66811 (5)	0.0245 (2)	
C12	0.55358 (10)	0.36401 (18)	0.64103 (5)	0.0253 (2)	
H12	0.5814	0.4682	0.6058	0.030*	
C13	0.64437 (10)	0.18317 (18)	0.66685 (5)	0.0243 (2)	
C14	0.60524 (11)	0.02991 (19)	0.71884 (5)	0.0263 (2)	
H14	0.6668	−0.0927	0.7358	0.032*	
C15	0.47284 (10)	0.06066 (19)	0.74563 (5)	0.0278 (2)	
H15	0.4454	−0.0420	0.7813	0.033*	
C16	0.38130 (11)	0.23721 (19)	0.72120 (5)	0.0270 (2)	
H16	0.2923	0.2562	0.7401	0.032*	
O13	0.77149 (8)	0.17389 (15)	0.63728 (4)	0.0316 (2)	
C17	0.86678 (11)	−0.0111 (2)	0.66159 (5)	0.0334 (3)	
H17A	0.8863	0.0179	0.7087	0.050*	
H17B	0.9532	−0.0014	0.6369	0.050*	
H17C	0.8257	−0.1758	0.6558	0.050*	

### Atomic displacement parameters (Å^2^)

**Table:**

	*U*^11^	*U*^22^	*U*^33^	*U*^12^	*U*^13^	*U*^23^
O1	0.0250 (4)	0.0310 (4)	0.0350 (4)	0.0067 (3)	0.0021 (3)	0.0095 (3)
C1	0.0255 (5)	0.0255 (5)	0.0277 (5)	0.0032 (4)	−0.0012 (4)	0.0029 (4)
C2	0.0262 (5)	0.0251 (5)	0.0281 (5)	0.0052 (4)	−0.0032 (4)	0.0004 (4)
C3	0.0290 (5)	0.0275 (5)	0.0294 (5)	0.0056 (4)	−0.0011 (4)	0.0014 (4)
C4	0.0291 (5)	0.0247 (5)	0.0268 (5)	0.0022 (4)	−0.0036 (4)	0.0007 (4)
O41	0.0362 (4)	0.0313 (4)	0.0351 (4)	0.0080 (3)	0.0025 (3)	0.0096 (3)
O42	0.0318 (4)	0.0371 (5)	0.0458 (5)	0.0105 (3)	0.0055 (4)	0.0148 (4)
C11	0.0238 (4)	0.0231 (4)	0.0266 (5)	0.0024 (3)	−0.0027 (4)	0.0014 (4)
C12	0.0263 (5)	0.0255 (5)	0.0240 (4)	0.0018 (3)	−0.0002 (3)	0.0035 (3)
C13	0.0237 (4)	0.0257 (4)	0.0235 (4)	0.0021 (3)	−0.0008 (3)	−0.0002 (4)
C14	0.0273 (5)	0.0250 (5)	0.0265 (5)	0.0018 (4)	−0.0033 (4)	0.0035 (4)
C15	0.0280 (5)	0.0282 (5)	0.0273 (5)	−0.0025 (4)	−0.0011 (4)	0.0050 (4)
C16	0.0245 (5)	0.0286 (5)	0.0280 (5)	−0.0010 (4)	0.0002 (4)	0.0020 (4)
O13	0.0266 (4)	0.0380 (4)	0.0303 (4)	0.0100 (3)	0.0048 (3)	0.0095 (3)
C17	0.0301 (5)	0.0382 (6)	0.0319 (5)	0.0128 (4)	0.0026 (4)	0.0059 (4)

### Geometric parameters (Å, °)

**Table:**

O1—C11	1.3747 (11)	C11—C16	1.4061 (14)
O1—C1	1.4343 (12)	C12—C13	1.4072 (13)
C1—C2	1.5219 (13)	C12—H12	0.9500
C1—H1A	0.9900	C13—O13	1.3732 (12)
C1—H1B	0.9900	C13—C14	1.3935 (14)
C2—C3	1.5263 (14)	C14—C15	1.4047 (14)
C2—H2A	0.9900	C14—H14	0.9500
C2—H2B	0.9900	C15—C16	1.3858 (14)
C3—C4	1.5103 (14)	C15—H15	0.9500
C3—H3A	0.9900	C16—H16	0.9500
C3—H3B	0.9900	O13—C17	1.4394 (12)
C4—O42	1.2217 (13)	C17—H17A	0.9800
C4—O41	1.3268 (13)	C17—H17B	0.9800
O41—H41	0.927 (18)	C17—H17C	0.9800
C11—C12	1.3977 (13)		
			
C11—O1—C1	118.38 (8)	O1—C11—C16	115.18 (9)
O1—C1—C2	106.92 (8)	C12—C11—C16	120.64 (9)
O1—C1—H1A	110.3	C11—C12—C13	118.96 (9)
C2—C1—H1A	110.3	C11—C12—H12	120.5
O1—C1—H1B	110.3	C13—C12—H12	120.5
C2—C1—H1B	110.3	O13—C13—C14	124.03 (9)
H1A—C1—H1B	108.6	O13—C13—C12	114.80 (8)
C1—C2—C3	110.58 (8)	C14—C13—C12	121.16 (9)
C1—C2—H2A	109.5	C13—C14—C15	118.56 (9)
C3—C2—H2A	109.5	C13—C14—H14	120.7
C1—C2—H2B	109.5	C15—C14—H14	120.7
C3—C2—H2B	109.5	C16—C15—C14	121.53 (9)
H2A—C2—H2B	108.1	C16—C15—H15	119.2
C4—C3—C2	113.88 (9)	C14—C15—H15	119.2
C4—C3—H3A	108.8	C15—C16—C11	119.14 (9)
C2—C3—H3A	108.8	C15—C16—H16	120.4
C4—C3—H3B	108.8	C11—C16—H16	120.4
C2—C3—H3B	108.8	C13—O13—C17	116.57 (8)
H3A—C3—H3B	107.7	O13—C17—H17A	109.5
O42—C4—O41	123.38 (9)	O13—C17—H17B	109.5
O42—C4—C3	124.16 (9)	H17A—C17—H17B	109.5
O41—C4—C3	112.46 (9)	O13—C17—H17C	109.5
C4—O41—H41	109.2 (11)	H17A—C17—H17C	109.5
O1—C11—C12	124.18 (9)	H17B—C17—H17C	109.5
			
C11—O1—C1—C2	−177.45 (8)	C11—C12—C13—C14	−0.36 (15)
O1—C1—C2—C3	−176.12 (8)	O13—C13—C14—C15	178.91 (9)
C1—C2—C3—C4	−174.52 (9)	C12—C13—C14—C15	−0.52 (15)
C2—C3—C4—O42	−5.13 (16)	C13—C14—C15—C16	0.51 (16)
C2—C3—C4—O41	174.73 (9)	C14—C15—C16—C11	0.38 (16)
C1—O1—C11—C12	−4.53 (15)	O1—C11—C16—C15	178.32 (9)
C1—O1—C11—C16	175.88 (9)	C12—C11—C16—C15	−1.28 (15)
O1—C11—C12—C13	−178.30 (9)	C14—C13—O13—C17	1.43 (15)
C16—C11—C12—C13	1.27 (15)	C12—C13—O13—C17	−179.11 (9)
C11—C12—C13—O13	−179.83 (9)		

### Hydrogen-bond geometry (Å, °)

**Table:**

*D*—H···*A*	*D*—H	H···*A*	*D*···*A*	*D*—H···*A*
O41—H41···O42^i^	0.927 (18)	1.804 (19)	2.7292 (11)	175.5 (16)
C17—H17B···O42^ii^	0.98	2.48	3.2477 (14)	135

Symmetry codes: (i) −*x*, −*y*+3, −*z*+1; (ii) *x*+1, *y*−1, *z*.
